# Interleukin-23 is critical for full-blown expression of a non-autoimmune destructive arthritis and regulates interleukin-17A and RORγt in γδ T cells

**DOI:** 10.1186/ar2893

**Published:** 2009-12-17

**Authors:** Ferry Cornelissen, Adriana MC Mus, Patrick S Asmawidjaja, Jan Piet van Hamburg, Joel Tocker, Erik Lubberts

**Affiliations:** 1 Department of Rheumatology, Erasmus Medical Center Rotterdam, Dr. Molewaterplein 50, Rotterdam, 3015 GE, the Netherlands; 2 Department of Immunology, Erasmus Medical Center Rotterdam, Dr. Molewaterplein 50, Rotterdam, 3015 GE, the Netherlands; 3 Amgen Inc., 51 University Street, Seattle, WA 98101-2918, USA

## Abstract

**Introduction:**

Interleukin (IL)-23 is essential for the development of various experimental autoimmune models. However, the role of IL-23 in non-autoimmune experimental arthritis remains unclear. Here, we examined the role of IL-23 in the non-autoimmune antigen-induced arthritis (AIA) model. In addition, the regulatory potential of IL-23 in IL-17A and retinoic acid-related orphan receptor gamma t (RORγt) expression in CD4^+ ^and TCRγδ^+ ^T cells was evaluated systemically as well as at the site of inflammation.

**Methods:**

Antigen-induced arthritis was induced in wild-type, IL-23p19-deficient and IL-17 Receptor A - knockout mice. At different time points, synovial cytokine and chemokine expression was measured. At days 1 and 7 of AIA, splenocytes and joint-infiltrating cells were isolated and analyzed for intracellular IL-17A and interferon (IFN)-γ *ex-vivo *by flow cytometry. In splenic CD4^+ ^and TCRγδ^+ ^T cells gene expression was quantified by flow cytometry and quantitative PCR.

**Results:**

IL-23 was critical for full-blown AIA. Lack of IL-23 did not prevent the onset of joint inflammation but stopped the progression to a destructive synovitis. IL-23 regulated IL-17A expression in CD4+ T cells in the spleen. Of note, IL-17A and IFN-γ expression was reduced in CD4^+ ^T cells in the inflamed joints of IL-23p19-deficient mice. Interestingly, IL-23 was also critical for the induction of IL-17A and RORγt but not IFN-γ in TCRγδ^+ ^T cells in the inflamed joints. The importance of the IL-23/IL-17 axis was further confirmed using IL-17 Receptor A knockout mice showing significantly milder AIA compared to control mice, with a disease course comparable to that of IL-23p19-deficient mice.

**Conclusions:**

These data show that IL-23 is critical for full-blown expression of a non-autoimmune destructive arthritis and regulates the proportion of IL-17A and IFN-γ-positive CD4^+ ^T cells at the site of inflammation. Furthermore, IL-23 regulates IL-17A and RORγt expression in TCRγδ T cells in arthritis. These findings indicate that regulating the IL-23 pathway may have therapeutic potential in non-autoimmune arthritis.

## Introduction

Interleukin (IL)-23 is a member of the IL-12 family and consists of both an IL-23-specific p19 subunit, and of a p40 subunit which is shared with IL-12 [1]. IL-23 is elevated in many autoimmune diseases such as psoriasis, rheumatoid arthritis (RA), multiple sclerosis (MS), and inflammatory bowel disease (IBD) [2]. It has been shown in animal models that IL-23, and not IL-12, is critical in the induction of autoimmunity [3-7]. Mice deficient for IL-23p19 were fully protected against collagen-induced arthritis (CIA), experimental autoimmune encephalomyelitis (EAE) and experimental autoimmune uveitis (EAU), in contrast to IL-12p35 deficient mice [3,4,6]. Although initial studies show that the development of autoimmunity was through Th17 cells, recent data show that also Th1 cells are able to induce pathology [6,8]. Thus, the role of Th17 and Th1 cells and their interaction in these models needs further clarification [9]. However, in all these autoimmune models it is evident that IL-23 is essential in their development. Still, it has not been elucidated whether IL-23 is critical for the progression of arthritis into a non-autoimmune destructive arthritis.

In collagen-immunized IL-23p19 knockout mice, no IL-17A-producing CD4^+ ^T cells were noted although no difference in IFN-γ-producing CD4^+ ^T cells was observed [4]. This indicates that IL-17A plays an important role in the early phase of CIA which is in line with earlier observations [10,11]. Apart from Th17 cells, other cells such as CD8^+^, NKT, and TCRγδ^+ ^T cells are able to produce IL-17A [12,13] and it has been shown that TCRγδ^+ ^T cells produced relatively high levels of IL-17A in CIA [14] and, in fact are the predominant source of IL-17-producing cells in the CIA joint [15]. Depletion of IL-17A-producing Vγ4^+ ^TCRγδ^+ ^T cells resulted in a significant reduction of the clinical disease score although mice were not fully protected [14]. However, the role of IL-23 in regulating IL-17A production in these TCRγδ^+ ^T cells is unknown.

Here, our results revealed that IL-23 is essential for the development of full-blown antigen-induced arthritis. We used IL-23p19 knockout (IL-23p19KO) mice to demonstrate that lack of IL-23 did not prevent the onset of joint inflammation but stopped the progression to a destructive synovitis. In the joints of IL-23p19KO mice, the proportions of IL-17A and IFN-γ-positive CD4^+ ^T cells were reduced. TCRγδ^+ ^T cells also required IL-23 for IL-17A but not for IFN-γ production in the inflamed joints. Of note, the transcription levels of RORγt were significantly higher in TCRγδ^+ ^T cells than in CD4^+ ^T cells from wild type mice. The importance of the IL-23/IL-17 axis was further confirmed using IL-17 Receptor A knockout (IL-17RAKO) mice showing a similar arthritis expression as IL-23p19KO mice. Thus, IL-23 is critical for full-blown expression of a non-autoimmune destructive arthritis. Furthermore, IL-23 regulates IL-17A and RORγt expression in TCRγδ^+ ^T cells during joint inflammation.

## Materials and methods

### Antigen-induced arthritis

IL-23p19 knockout mice were kindly provided by Dr. N. Ghilardi, Genentech Inc., San Francisco, CA, USA [16], and IL-17 Receptor A knockout mice by Dr. J. Tocker, Amgen Inc., Seattle, WA, USA [17]. Both strains were backcrossed on the C57BL/6 background for at least 10 generations. Mice were kept under specific pathogen free conditions and provided with food and water *ad libitum*. Mice between 8 and 12 weeks of age were used for experiments. All experiments were approved by the Dutch Animal Ethics Committee (DEC).

To induce AIA, methylated Bovine Serum Albumin (mBSA, 8 mg/mL) was emulsified in an equal volume of CFA containing 1 mg/mL heat-killed *M. tuberculosis *(H37Ra; Difco). At day 7, mice were immunized intradermal with 100 μL mBSA/CFA. One week later, 60 μg mBSA was injected intra-articular to induce mono-arthritis. The severity of arthritis in the knee joint was scored macroscopically on a scale of 0 to 2 after removing the skin. At different time points during AIA, rear limbs were hematoxylin and eosin stained as previously described [18], and the severity of joint infiltration and bone erosion was determined on a scale from 0 to 3 (0 = no infiltration, 1 = mild infiltration, 2 = moderate infiltration, 3 = maximal infiltration; 0 = no erosion, 1 = mild erosion, 2 = moderate erosion and 3 = maximal erosion).

### Synovial cytokine levels

To measure synovial cytokine levels, patellae with adjacent synovium was isolated from knee joints as described earlier [18]. MCP-1, TNF-α, IL-6, IFN-γ, IL-12p70 and IL-10 were measured using the cytometric bead array using the mouse inflammation kit with a detection limit of 10 pg/ml (BD Biosciences, Sunnyvale, CA, USA). I IL-17A was measured by ELISA (R&D Systems, Minneapolis, MN, USA).

### Single-cell isolation and flow-cytometric analyses

With Blendzyme3 (60 ug/ml, Roche Diagnostics, Mannheim, Germany), cells from inflamed joints were isolated [18]. For intracellular detection of cytokines, we stimulated splenocytes or cells isolated from the joints with phorbol myristate acetate (PMA) (0.05 μg/mL) and Ionomycin (0.5 μg/mL) in the presence of GolgiStop™ (BD Biosciences) for four hours. For intracellular cytokine staining, cells were fixed using 2% PFA and permeabilized in 0.5% saponin. For intracellular staining of RORγt, T-bet and GATA-3, a Fixation and Permeabilization kit (eBioscience, San Diego, CA, USA) was used. Samples were acquired on a FACS Calibur or on a FACS CANTO flow cytometer and analyzed using FlowJo (Tree Star, Inc., Ashland, OR, USA) software.

### Purification of effector t cells and in vitro t cell stimulation

CD3^+^CD4^+ ^and CD3^+^TCRγδ^+ ^T cells were FACS-sorted from spleens obtained at day 7 of AIA using a FACS Aria cell sorting system and BD FACS Diva software (BD Bioscience). Purity of obtained fractions was >98%.

### Quantitative PCR analyses

Total RNA of sorted CD3^+^CD4^+ ^and CD3^+^TCRγδ^+ ^T cells was extracted, and DNaseI-treated RNA was used for cDNA synthesis [18]. PCR primers were designed manually or using ProbeFinder software (Roche Applied Science, Indianapolis, IN, USA) and probes were chosen from the universal probe library (Roche Applied Science). Quantitative realtime PCR was performed using the ABI Prism 7900 HT sequence-detection system (Applied Biosystems, Foster City, CA, USA) and analyzed using SDS v2.3 software (Applied Biosystems). The Ct values obtained were normalized to those of glycereraldehyde-3-phosphate dehydrogenase (GAPDH).

### Statistical analysis

Differences between groups were tested with the Mann-Whitney *U *test or the unpaired student t-test. *P *values less than 0.05 were considered significant.

## Results

### IL-23 has a critical role in the progression of destructive arthritis

**Figure 1:**
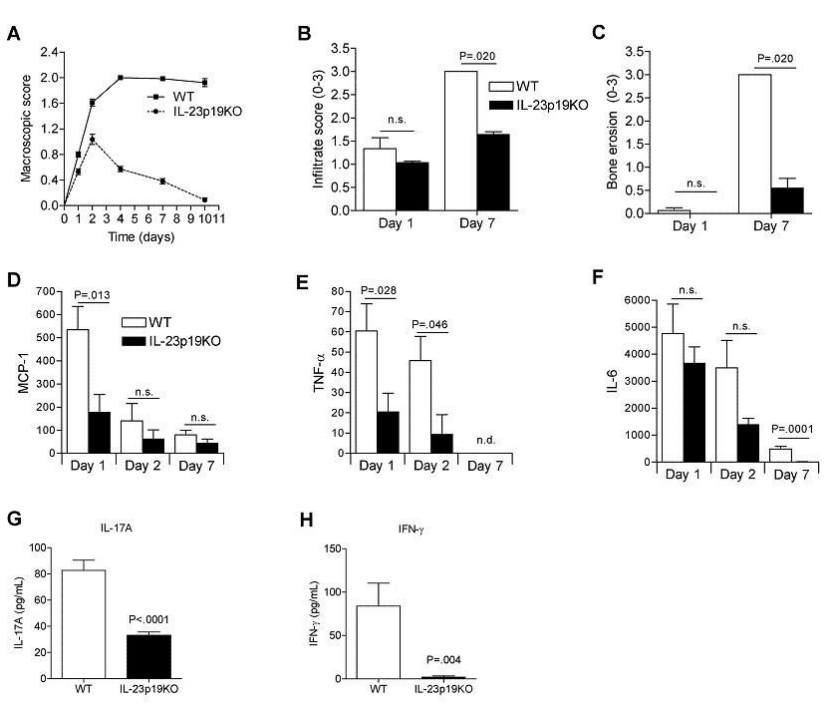
IL-23 has a critical role in the progression of antigen-induced arthritis. WT and IL-23p19KO mice were immunized with mBSA/CFA and one week later mono-arthritis was induced by injecting mBSA directly into the knee joints. **A**. Arthritis score was determined macroscopically at different time points. Mean values and SEM are given for 8 to 31 mice per group. Data are obtained from three separate experiments. * *P *< 0.001, WT vs IL-23p19KO. **B**, Histological analyses of joint inflammation and, **C**, bone erosion after H&E staining. *B *and *C*, Mean values and SEM are given from two separate experiments with a total of 20 knee joints per group. **D-F**. Cytokine levels in synovial washouts taken at days 1, 2 and 7. **G and H**. IL-17A and IFN-γ levels in synovial washouts taken at day 1. Mean values and SEM are given for 5 to 10 washouts obtained from two separate experiments.

To investigate the role of IL-23 in the progression of a T cell mediated destructive inflammatory arthritis, we induced antigen-induced arthritis (AIA) in WT and IL-23p19 knockout (IL-23p19KO) mice by immunization with methylated BSA (mBSA) in Complete Freund's Adjuvant (CFA) and induced mono-arthritis one week later by a single intra-articular injection of mBSA into the knee joint. Maximum arthritis score was observed at day 4, which stayed high until day 10 (Figure 1A). In contrast, in IL-23p19KO mice the onset of arthritis was not prevented although significantly milder joint inflammation was noted which decreased rapidly to almost normal at day 10. Seven days after the induction of mono-arthritis, histological analyses revealed significantly less joint infiltration (Figure 1B) and bone erosion (Figure 1C) in IL-23p19KO mice than in WT mice. These data show that IL-23 is required for the progression of AIA into destructive synovitis.

To gain insight in the local chemokine and cytokine expression, synovial washouts from WT and IL-23p19KO mice were taken at different time points during AIA. The highest expression of MCP-1 was measured at day 1 which was significantly lower in IL-23p19KO compared to WT mice (Figure 1D). TNF-α was significantly lower in IL-23p19KO than in WT mice at days 1 and 2, and undetectable at day 7 (Figure 1E). Also IL-6 was measured in synovial washouts but no statistical significant differences of this cytokine were observed between WT and IL-23p19KO mice at days 1 and 2 (Figure 1F). Of high interest, IL-17A and IFN-γ levels were significantly lower in IL-23p19KO than in WT mice at day 1 of AIA (Figure 1G and 1H). Of note, no IL-12p70 and IL-10 could be detected (data not shown).

### The induction of Th17 cells in antigen-induced arthritis is IL-23 dependent

**Figure 2:**
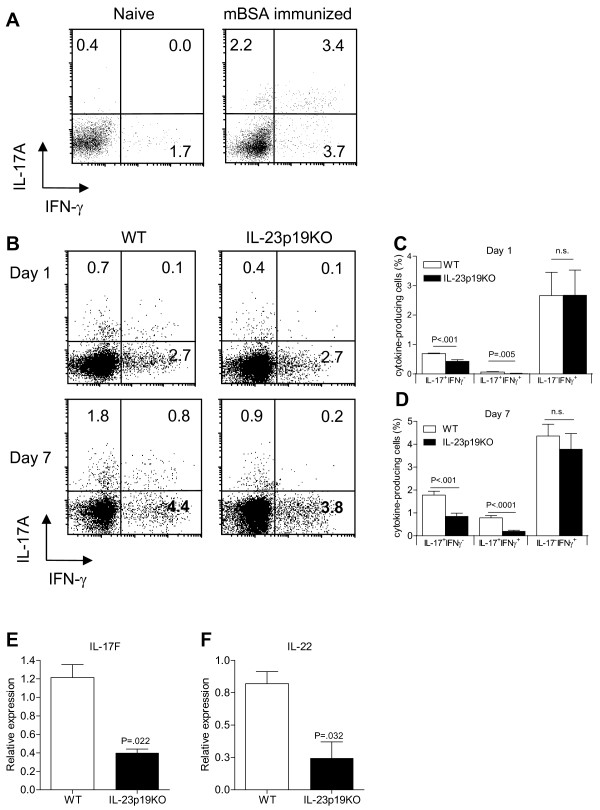
The induction of Th17 cells in AIA is IL-23 mediated. **A**. WT mice were immunized with mBSA/CFA and 10 days later splenocytes were isolated and stimulated for four hours with PMA/Ionomycin, gated for CD4^+ ^T cells and analyzed for intracellular IL-17A and IFN-γ expression. Numbers indicate percentage of positive cells within each quadrant. **B-D**. Antigen-induced arthritis was induced in WT and IL-23p19KO mice and at days 1 and 7, after i.a. mBSA injection, the splenocytes were isolated and stimulated for 4 h with PMA/Ionomycin and analyzed for intracellular IL-17A and IFN-γ expression on a CD4^+ ^T cell gate. B. Numbers indicate percentage of positive cells within each quadrant. C and D. Quantification of flow cytrometric analyses from B; mean values and SEM are given for 6 to 12 mice per group from two to four independent experiments. **E and F**. On day 7 of AIA, splenic CD3^+^CD4^+ ^T cells were FACS-sorted and gene expression was analyzed by quantitative RT-PCR for IL-17F and IL-22 respectively. Mean values and SEM are given for three mice. *P*-values were calculated using the student t-test.

To examine whether Th17 cells are induced in AIA, WT mice were immunized with mBSA/CFA. Ten days after immunization, the proportions of IL-17A^+^IFN-γ^-^, IL-17A^+^IFN-γ^+ ^*double positive *and IL-17A^-^IFN-γ^+ ^CD4^+ ^T cells were increased compared to naïve mice (Figure 2A) indicating the induction of both Th17 and Th1 cells in AIA.

Because the arthritis progression was IL-23 dependent, we further examined the role of IL-23 on the formation of IL-17A and IFN-γ-producing CD4^+ ^T cells in this process. Splenocytes were isolated from arthritic WT and IL-23p19KO mice at days 1 and 7 of AIA. At both time points, the proportions of IL-17A^+^IFN-γ^- ^and IL-17A^+^IFN-γ^+ ^*double positive *CD4^+ ^T cells were lower in IL-23p19KO than in WT mice, while the proportion of IL-17A^-^IFN-γ^+ ^CD4^+ ^T cells was similar between both groups (Figure 2B-D; Figure S1 in Additional file 1 shows the FSC/SSC of total splenocytes from WT and IL-23p19KO mice). In addition to the decrease of IL-17A-producing cells, a significant reduction in IL-17F and IL-22 mRNA expression was found in FACS-sorted splenic CD4^+ ^T cells (Figure 2E-F).

### IL-23 is essential for the induction of IL-17A and RORγt in TCRγδ^+ ^T cells during arthritis

**Figure 3:**
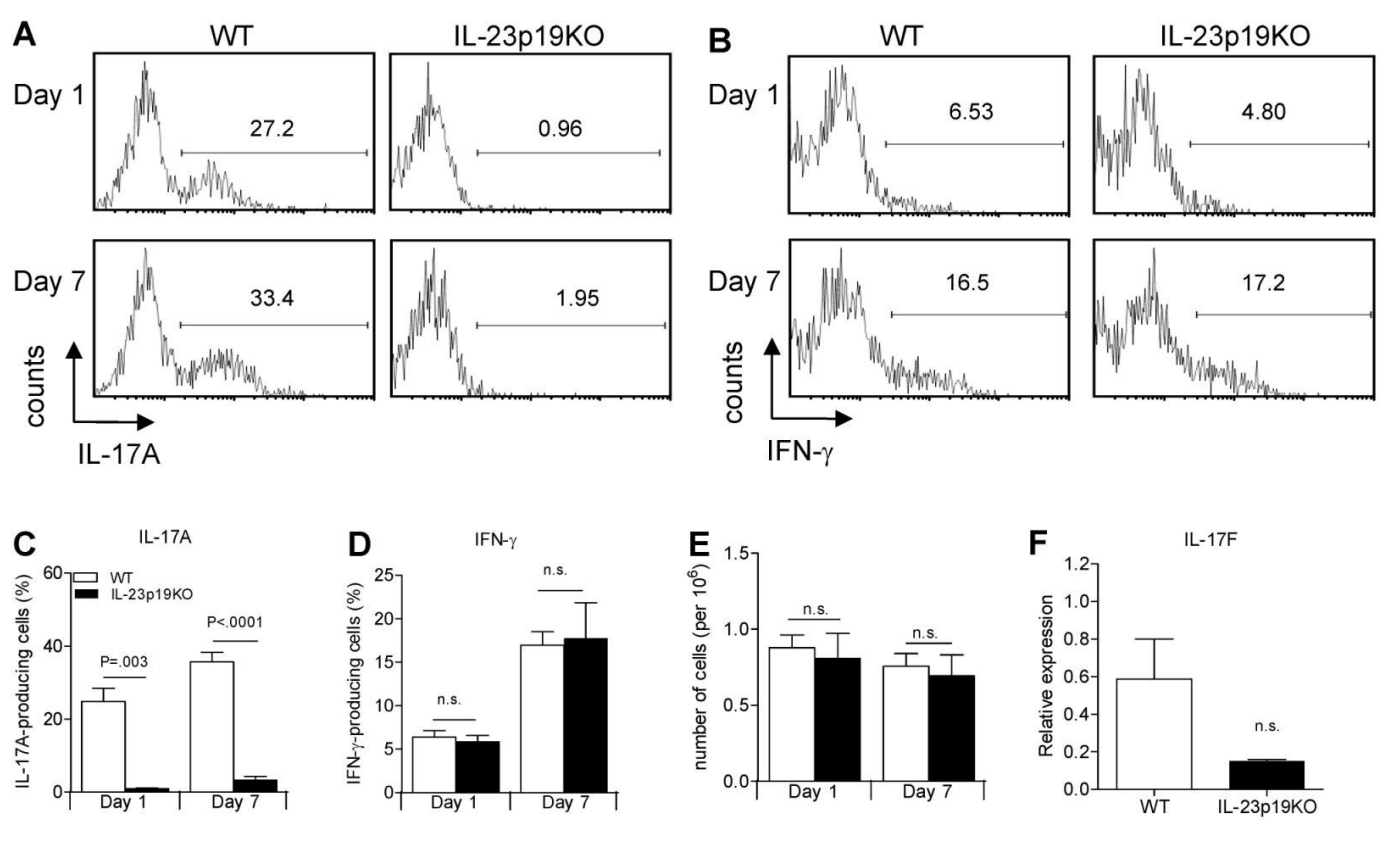
IL-23 is critical for the induction of IL-17A and RORγt in TCRγδ T cells. **A **and **B**. At days 1 and 7 of AIA, splenocytes were isolated and stimulated for four hours with PMA/Ionomycin, gated for CD3^+^TCRγδ^+ ^T cells and analyzed for intracellular IL-17A and IFN-γ respectively. Numbers in quadrants indicate percentage of cytokine-positive cells. **C **and **D**. Quantification of flow cytrometric analyses from *A *and *B *for IL-17A and IFN-γ; mean values and SEM are given for three to six mice for day 1 and for nine mice for day 7. **E**. Cell counts of total amount of TCRγδ^+ ^T cells present in spleen on day 1 and 7 of AIA in WT and IL-23p19KO mice. **F**. On day 7 of AIA, splenic CD3^+^TCRγδ^+ ^T cells were FACS-sorted and IL-17F gene expression was analyzed by quantitative RT-PCR. Mean values and SEM are given for three mice. *P*-values were calculated using the student's t-test.

To investigate whether TCRγδ^+ ^T cells were present during AIA and able to produce IL-17A as was described for CIA [14], we isolated splenocytes at days 1 and 7 of AIA. On day 1, a relatively high proportion of TCRγδ^+^IL-17A^+ ^T cells was detected in the spleen of WT mice, and this proportion was elevated at day 7 (Figure 3A and 3C). Interestingly, on these time points, a significantly lower proportion of TCRγδ^+^IL-17A^+ ^T cells was present in IL-23p19KO than in WT mice (Figure 3A and 3C), which was not associated with lower numbers of splenic TCRγδ^+ ^T cells (Figure 3E). Of note, the proportion of TCRγδ^+^IL-17A^+ ^T cells was 15- to 27-fold reduced in IL-23p19KO mice versus WT mice (Figure 3A and 3C) compared to a two-fold reduction of CD4^+^IL-17A^+ ^T cells (Figure 2B-D). The proportions of both IL-17A-positive TCRγδ^+ ^and CD4^+ ^T cells isolated from the draining lymph-nodes were substantially reduced in IL-23p19KO mice versus WT mice (Figure S3 in Additional file 1). Notably, since the proportion of IL-17A-positive CD4^+ ^and TCRγδ^+ ^T cells were not decreased in splenocytes taken from naïve IL-23p19KO compared to naïve WT mice, we can exclude that there is an intrinsic deficiency of PMA/Ionomycin-induced IL-17A-production in IL-23p19KO cells (Figure S2 in Additional file 1). These data indicate that the IL-17A production in TCRγδ^+ ^T cells during AIA is highly IL-23 dependent.

Apart from IL-17A, we analyzed the production of IFN-γ by TCRγδ^+ ^T cells in WT and IL-23p19KO mice. From day 1 to day 7, the proportion of TCRγδ^+^IFN-γ^+ ^T cells was equally increased in WT and IL-23p19KO mice (Figure 3B and 3D) suggesting that IFN-γ production by TCRγδ^+ ^T cells is independent of IL-23.

Because it is not known whether the expression of IL-17F and IL-22 in TCRγδ^+ ^T cells is IL-23 mediated *in vivo*, we performed quantitative PCR on FACS-sorted splenic TCRγδ^+ ^T cells from WT and IL-23p19KO mice isolated at day 7 of AIA. Although we could not detect IL-22 mRNA; the expression of IL-17F was markedly lower in IL-23p19KO TCRγδ^+ ^T cells compared to WT TCRγδ^+ ^T cells (Figure 3F).

### IL-23 regulates RORγt, but not T-bet, in TCRγδ^+ ^T cells

**Figure 4:**
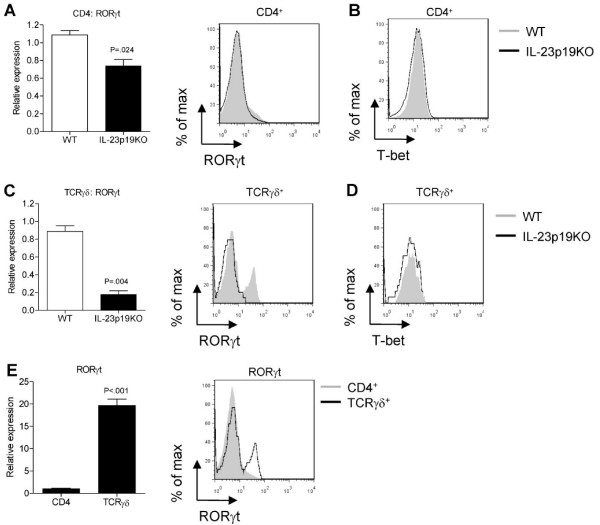
IL-23 is essential for RORγt expression in TCRγδ ^+ ^T cells. At day 7 of AIA, WT and IL-23p19KO mice were sacrificed and splenocytes were isolated. **A**. CD3^+^CD4^+ ^T cells were FACS-sorted and RORγt gene expression was analyzed by quantitative RT-PCR (left panel); RORγt was measured intracellular and CD4^+ ^T cells were gated (right panel). **B **T-bet expression was measured intracellular and CD4^+ ^T cells were gated. **C**. CD3^+^TCRγδ^+ ^T cells were FACS-sorted and RORγt gene expression was analyzed by quantitative RT-PCR (left panel); RORγt was measured intracellular and γδ^+ ^T cells were gated (right panel). **D**. T-bet expression was measured intracellular and γδ^+ ^T cells were gated. **E**. Comparison of the mRNA quantification of RORγt expression between FACS-sorted CD4^+ ^and γδ^+ ^T cells. Mean values and SEM are given for three mice and *P*-values were calculated using the student's t-test.

Since the proportion of CD4^+ ^IL-17A^+^IFN-γ^- ^but not IL-17A^-^IFN-γ^+ ^T cells was significantly lower in IL-23p19KO than in WT mice, we measured RORγt mRNA expression in FACS-sorted splenic CD4^+ ^and TCRγδ^+ ^T cells isolated at day 7 of AIA. In addition, RORγt, T-bet and GATA3 expression was measured by intracellular protein-stainings. No GATA3 expression by flow cytometry was found in neither CD4^+ ^nor in TCRγδ^+ ^T cells (data not shown). Figure 4A shows that RORγt was decreased in CD4^+ ^T cells from IL-23p19KO mice compared to WT mice, while T-bet expression was similar (Figure 4B). In TCRγδ^+ ^T cells, the expression of RORγt was also significantly reduced by lack of IL-23p19 (Figure 4C), while T-bet expression was similar between WT and IL-23p19KO γδ T cells (Figure 4D). Interestingly, RORγt was expressed at higher levels in WT TCRγδ^+ ^T cells than in WT CD4^+ ^T cells (Figure 4E).

### IL-23 is essential for local IL-17A-production by TCRγδ^+ ^and CD4^+ ^T cells

**Figure 5:**
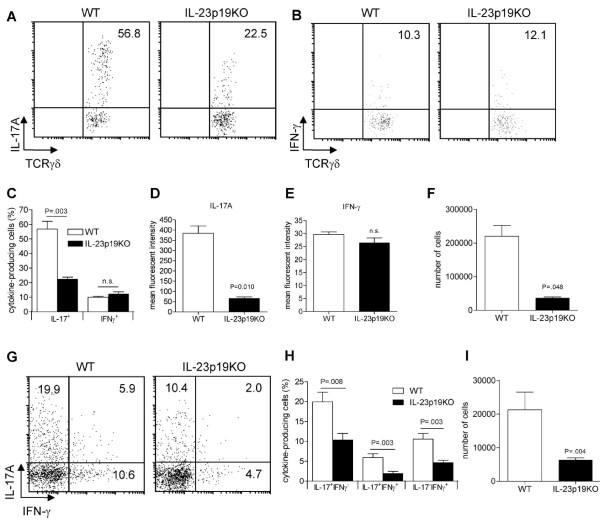
IL-23 deficiency results in less IL-17 production in the inflamed joint. At day 7 of AIA, cells from the arthritic joints of WT and IL-23p19KO mice were isolated and stimulated for four hours with PMA/Ionomycin. **A**. Intracellular cytokine staining of TCRγδ^+ ^T cells for IL-17. **B**. Intracellular cytokine staining of TCRγδ^+ ^T cells for IFN-γ; data from *A *and *B *are representatives from three mice per group. **C**. Quantification of flow cytrometric analyses from *A *and *B*. **D**. MFI was calculated for TCRγδ^+ ^IL-17^+ ^T cells. **E**. MFI was calculated for TCRγδ^+ ^IFN-γ^+ ^T cells. **F**. Total numbers of TCRγδ^+ ^T cells in the joints of arthritic WT and IL-23p19KO mice. *C-F*, Mean values and SEM are given for three mice and *P*-values were calculated using the student t-test. **G**. Intracellular cytokine staining of CD4^+ ^T cells for IL-17A and IFN-γ. Data are representatives of six mice per group. **H**. Quantification of flow cytrometric analyses from *G*. **I**. Total numbers of CD4^+ ^T cells present in arthritic joints from WT and IL-23p19KO mice.

Since severe joint inflammation was observed in WT but not in IL-23p19KO mice at day 7 of AIA (Figure 1A), we wondered whether the proportions of IL-17A-producing cells in the inflamed joints of WT and IL-23p19KO mice were different. A significantly lower proportion of IL-17A, but not of IFN-γ-producing TCRγδ^+ ^T cells was observed in the inflamed joints of IL-23p19KO mice compared to WT mice (Figure 5A-C; Figure S4 in Additional file 1 shows the gating-strategy we used to plot TCRγδ^+ ^T cells isolated from the joint). Interestingly, the mean-fluorescent intensity (MFI) of WT IL-17A-producing TCRγδ^+ ^T cells was significantly higher than the MFI of these cells in IL-23p19KO (Figure 5D). In contrast, the MFI of IFN-γ in the IFN-γ-producing TCRγδ^+ ^T cells was similar between IL-23p19KO and WT controls (Figure 5E). In line with the reduced level of inflammation observed in IL-23p19KO mice (Figure 1B), the total number of TCRγδ^+ ^T cells was lower in the arthritic joints from IL-23p19KO mice than from WT mice (Figure 5F).

At the site of inflammation, the proportions of CD4^+ ^IL-17A^+^IFN-γ^-^, IL-17A^+^IFN-γ^+ ^*double positive *and IL-17A^-^IFN-γ^+ ^T cells were markedly elevated compared to the spleen (Figures 2B-D and 5G-H). In addition, in IL-23p19KO mice, the absolute numbers of CD4^+ ^T cells were significantly lower compared to WT mice (Figure 5I).

### Critical role for the IL-23/IL-17 immune pathway in the progression of destructive arthritis

**Figure 6:**
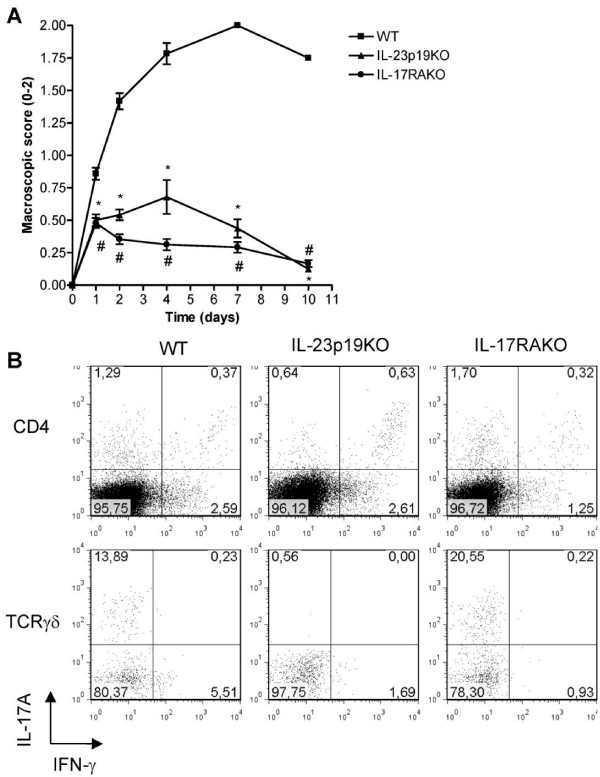
IL-17 receptor signaling is critical for the progression of AIA. **A**. WT, IL-23p19KO and IL-17RAKO mice were immunized with mBSA/CFA and mono-arthritis was induced one week later by an i.a. injection of mBSA. At days 1, 2, 4, 7 and 10 macroscopic scores were assessed. Mean values and SEM are given for six mice per group. * *P *< 0.01 WT vs IL-23P19KO and ^#^, *P *< 0.01 WT vs IL-17RAKO. **B**. At day 7 of AIA, splenocytes from WT, IL-23p19KO and IL-17RAKO were isolated and stimulated for four hours with PMA/Ionomycin and analyzed for intracellular IL-17A and IFN-γ expression on a CD4^+ ^and TCRγδ^+ ^T cell gate. Numbers indicate percentage of positive cells within each quadrant and each dot plot shows a representative mouse from three mice per group analyzed.

Since the proportions and numbers of IL-17A but also of IFN-γ-producing CD4^+ ^T cells in the inflamed joint were lower in IL-23p19KO than in WT mice (Figure 5G-I), we investigated the importance of IL-17A signaling using IL-17 receptor A knockout (IL-17RAKO) mice. IL-17RAKO mice showed a similar pattern of arthritis as IL-23p19KO mice did, and this was significantly suppressed in both mouse knockout strains compared to WT mice (Figure 6A). The lack of IL-17R-signalling did however not lead to a reduced proportion of IL-17A-positive CD4^+ ^or TCRγδ^+ ^T cells (Figure 6B).

## Discussion

Our findings show for the first time that IL-23 is critical for full-blown non-autoimmune antigen-induced arthritis. IL-23 deficiency did not prevent the onset of joint inflammation but stopped the progression to a destructive synovitis. In the inflamed joint, IL-17A and IFN-γ-producing CD4^+ ^T cells were reduced under IL-23-deficient conditions. In addition, IL-23 was also essential for the induction of IL-17A and RORγt, but not for IFN-γ, in TCRγδ^+ ^T cells both systemically and locally at the site of inflammation. The importance of the IL-23/IL-17 immune pathway was confirmed by using IL-17RAKO mice which developed arthritis similar to IL-23p19KO mice. These data show that IL-23 is critical in the regulation of a non-autoimmune inflammatory arthritis and that it regulates IL-17A and RORγt expression in CD4^+ ^and TCRγδ^+ ^T cells.

IL-23 is required for Th17 function *in vivo *and is considered to be a survival factor for these T cells [19,20]. TGF-β and IL-6 in contrast to IL-23 are critical in Th17 polarization from naïve T cells [21-23]. Interestingly, TGF-β and IL-6 drive the production of IL-17A and IL-10 by T cells and restrain Th17 cell-mediated pathology [24]. In addition, IL-23 promotes the proinflammatory Th17 profile induced by TGF-β and IL-6 and is required for IL-22 expression [[25,26] and Lubberts et al. unpublished observations]. In the present study, we found that IL-23 deficiency did not prevent the onset of antigen-induced arthritis. Although MCP-1, TNF-α, IL-17A and IFN-γ as well as joint inflammation were suppressed in the early phase of arthritis in IL-23p19KO compared to WT mice, these data indicate that the early joint inflammatory responses in this non-autoimmune T cell mediated arthritis-model is at least partly IL-23 independent. In contrast, further progression of arthritis into a chronic destructive arthritis is IL-23 dependent. In this stage of the disease effector T cells migrate from the lymphoid tissues to the inflamed joint and play an important role in the progression of arthritis. Whether this is solely a matter of Th17 cells or that other IL-17A producing cells are involved as well needs further investigation, especially since we now found that IL-23 regulates IL-17A production in TCRγδ^+ ^T cells.

In addition to Th17 cells there are other IL-17A-producing T cells such as CD8^+^, NKT and TCRγδ^+ ^T cells [27-31]. A direct role for TCRγδ^+ ^T cells in the pathogenesis of collagen-induced arthritis has been demonstrated [32]. Interestingly, these cells produced relatively high levels of IL-17A [14]. In fact it has been shown that the number of IL-17-producing Vγ4^+ ^TCRγδ^+ ^T cells in the draining lymph nodes was equal to the number of CD4^+ ^TCRαβ^+ ^Th17 cells [14] and, most recently, it has been shown that the predominant IL-17-producing T cells in the joint of CIA-mice are TCRγδ^+ ^and not CD4^+ ^[15]. Depletion of Vγ4^+ ^TCRγδ^+ ^T cells significantly reduced clinical disease scores and incidence of disease [14]. On the other hand, depletion of TCRγδ^+ ^T cells did not prevent or ameliorate but rather aggravate rat adjuvant arthritis [33]. Here, we found a relatively high percentage of IL-17-producing TCRγδ^+ ^T cells in the spleen and inflamed joints of arthritic wild type mice. Of note, in contrast to IL-17A and IL-17F, no expression of IL-22 was detected in FACS-sorted splenic TCRγδ^+ ^T cells from WT arthritic mice. Of high interest, IL-23 regulates IL-17A production in TCRγδ^+ ^T cells in spleen, lymph nodes as well as in the inflamed joints. In contrast, no reduction in the proportion of IFN-γ-producing TCRγδ^+ ^T cells was noted in IL-23p19KO mice compared to WT mice. These data show for the first time that the formation of IL-17A and RORγt-expressing TCRγδ^+ ^T cells is IL-23 dependent *in vivo*. However, this study does not reveal the contribution of IL-17A-producing TCRγδ^+ ^T cells in the arthritis process.

Th17 differentiation is driven by the orphan nuclear receptor RORγt [34] and RORα [35]. We found that, compared to CD4^+ ^T cells, the expression of RORγt was substantially higher in TCRγδ^+ ^T cells, which corresponds with the higher proportion of IL-17A-producing cells in the TCRγδ^+ ^fraction than in the CD4^+ ^T cell pool. This suggests that in TCRγδ^+ ^T cells, as in Th17 cells, the production of IL-17A and perhaps also of IL-17F is under control of RORγt, which is in line with earlier observations in which it has been shown that in lung and skin the largest population of RORγt^+ ^T cells express the γδ TCR and produce the highest levels of IL-17A [36]. In the present study, a clear reduction of RORγt levels in IL-23p19KO mice under arthritic conditions was observed. This suggests that IL-23 regulates the induction of RORγt in γδ T cells.

Next to regulation of CD4^+ ^IL-17^+^IFN-γ^- ^T cells by IL-23, data from the present study also show marked reduction of CD4^+ ^IL-17^+^IFN-γ^+ ^*double positive *cells in the absence of IL-23 during arthritis. The role and function of these *double positive *cells in the inflammatory process is unknown. Since these cells express both IL-17 and IFN-γ reflecting both Th17 and Th1 cytokine activity, these cells might be even more pathogenic than single IL-17^+ ^or IFN-γ^+ ^T cells. On the other hand, these cells may represent the transition phase of Th17 into Th1 cells. It has been shown that IFN-γ is protective against bone erosion and the expression of IFN-γ in human arthritis is accompanied with less joint destruction [37,38]. This might indicate that the IL-17^+^IFN-γ^+ ^*double positive *cells might be less pathogenic compared to IL-17^+^IFN-γ^- ^T cells in arthritis. Although Th17 cells are more pathogenic in inducing autoimmune diseases than IFN-γ producing Th1 cells in mice [3,4,6], it was shown that both Th1 and Th17 cells are able to induce EAE after adaptive T cell transfer of these specific Th subsets into nude mice although Th1 and Th17 cells induced different pathology [8]. In this study we showed that the IL-23/IL-17A immune pathway is critical for the progression of arthritis into a chronic destructive synovitis in a non-autoimmune arthritis model, which is in line with the critical pathogenic role for IL-17A in the arthritis process [39]. Elevated levels of the pro-inflammatory T cell cytokine IL-17A have been detected in synovia of RA patients[40,41]. IL-17A contributes to the pathogenesis of destructive arthritis [42,43]. Important sources of IL-17A in this disease are Th17 cells and potentially TCRγδ^+ ^T cells. This latter cell type was elevated in synovia from RA patients with active synovitis and those RA patients with increased synovial TCRγδ^+ ^T cells had an increased tissue inflammation score compared to RA synovia with few TCRγδ^+ ^T cells [44]. However, this study does not show the dominance of TCRγδ^+ ^T cells compared with conventional TCRαβ T cells. In collagen-induced arthritis, it has been shown that a relatively high proportion of TCRγδ^+ ^T cells are able to produce IL-17A [14] and are present at relatively high cell numbers in CIA joints [15].

In contrast, in the synovia of patients with established RA, only few IL-17-producing TCRγδ T cells were present [15]. Therefore, studies on early RA patient materials including synovial tissue infiltrates are needed to evaluate the presence of IL-17-producing TCRγδ T cells. Interestingly, CD4^+^CD45RO^+^IL-17A^+ ^T cells were found in treatment-naïve early RA patients with active disease [45]. The present observation that IL-23 regulates IL-17A production in both Th17 cells and TCRγδ T cells in experimental arthritis underscores the need for further studies to unravel the potential of IL-23 as a therapeutic target in the pathogenesis of human destructive arthritis.

In conclusion, this study shows that IL-23 is critical for full-blown expression of a non-autoimmune destructive arthritis. Furthermore, IL-23 regulates the formation of both CD4^+ ^and TCRγδ^+ ^IL-17A-producing T cells. These data add new insight to the role of IL-23 in the regulation of a non-autoimmune inflammatory arthritis. Furthermore, these data show that IL-23 regulates IL-17A and RORγt expression in TCRγδ^+ ^T cells during joint inflammation. These findings may be relevant to other chronic inflammatory conditions and infectious diseases as well.

## Conclusions

The aim of our study was to examine the role of IL-23 in the non-autoimmune antigen-induced arthritis model. Additionally, we investigated the regulatory potential of IL-23 in IL-17A and RORγt expression in CD4^+ ^and TCRγδ^+ ^T cells from the spleen and joints of arthritic mice. In the present study we showed that IL-23 is essential for the development of full-blown antigen-induced arthritis; IL-23p19-deficiency did not prevent the onset of joint inflammation but stopped the progression to a destructive synovitis. Furthermore, in the inflamed joints of IL-23p19KO mice, the proportion of IL-17A and IFN-γ-expressing CD4^+ ^T cells were reduced whereas IL-23 was also required for IL-17A but not for IFN-γ-producing TCRγδ^+ ^T cells in the inflamed joints. The transcription levels of RORγt were significantly higher in TCRγδ^+ ^T cells than in CD4^+ ^T cells from wild type arthritic mice. Finally, since CD4^+^IFN-γ^+ ^cells were lower in the inflamed joints of IL-23p19-deficient mice, we confirmed the importance of the IL-23/IL-17 axis using IL-17RA deficient mice showing a similar arthritis expression as IL-23p19KO mice.

Thus, this study adds new insight to the role of IL-23 in the regulation of non-autoimmune arthritis. Furthermore, although it is suggested that IL-23 is involved in IL-17A-production by TCRγδ^+ ^T cells, we show for the first time a direct *in vivo *role for IL-23 in regulating IL-17A and RORγt expression by TCRγδ^+ ^cells. These findings indicate that regulating the IL-23 pathway may have therapeutic potential in non-autoimmune arthritis.

## Abbreviations

AIA: antigen-induced arthritis; CFA: Complete Freund's Adjuvant; CIA: collagen-induced arthritis; mBSA: methylated Bovine Serum Albumin; MFI: Mean Fleuorescent Intensity; RA: rheumatoid arthritis; TCR: T cell receptor.

## Competing interests

The authors declare that they have no competing interests.

## Authors' contributions

FC participated in the design of the study, performed the experiments, collected and statistically analyzed the data, and drafted the manuscript. AMCM participated in the animal experiments and in collecting the data. PSA participated in the animal experiments and in collecting the data. JPvH participated in the design of the study and helped to draft the manuscript. JT participated in the design of the study and helped to draft the manuscript. EL conceived of the study, and was responsible for the design and coordination of the study and writing of the manuscript. All authors read and approved the final manuscript.

## Supplementary Material

Additional file 1 **An Adobe PDF file containing four supplemental figures**. **Figure S1**: Antigen-induced arthritis was induced in WT and IL-23p19KO mice and at day 7 after i.a. mBSA injection the splenocytes were isolated and stimulated for four hours with PMA/Ionomycin and analyzed by flow cytometry. Shown are total ungated cells. **Figure S2**: Splenocytes were isolated from naïve WT and IL-23p19KO mice and stimulated for four hours with PMA/Ionomycin and analyzed by flow cytometry for intracellular expression of IL-17A and IFN-γ. CD4^+ ^(top panel) and CD3^+^TCRγδ^+ ^(lower panel) T cells were gated. Numbers indicate percentage of cytokine-positive cells within each quadrant. **Figure S3**: Antigen-induced arthritis was induced in WT and IL-23p19KO mice and at day 7 after i.a. mBSA injection cells from the draining lymph-nodes were isolated and stimulated for four hours with PMA/Ionomycin and analyzed by flow cytometry. CD4^+ ^(top panel) and CD3^+^TCRγδ^+ ^(lower panel) T cells were gated. Numbers indicate percentage of positive cells within each gate. **Figure S4**: Pseudocolor plot of cells isolated from the joint of a representative WT mouse at day 7 of AIA. Shown is the FSC/SSC of all cells (left figure), and subsequent gating-steps for plotting TCRγδ^+ ^T cells. Numbers adjacent to gates indicate the percentage of cells in that specific gate and the numbers in the top-right corner indicate the percentage of cells in the gate relative to all cells. Click here for file
