# Supplementary figures and images for: Interleukin-23 is critical for full-blown expression of a non-autoimmune destructive arthritis and regulates interleukin-17A and RORγt in γδ T cells
Source: Arthritis Res Ther. 2009 Dec 17;11(6):R194. doi: 10.1186/ar2893 (PMC3003524; doi:10.1186/ar2893)

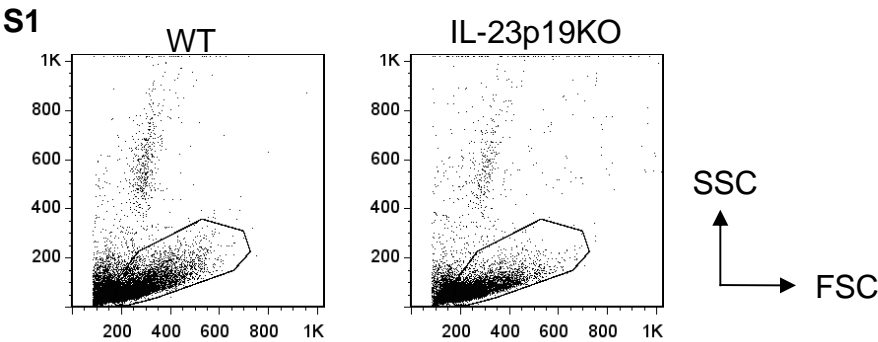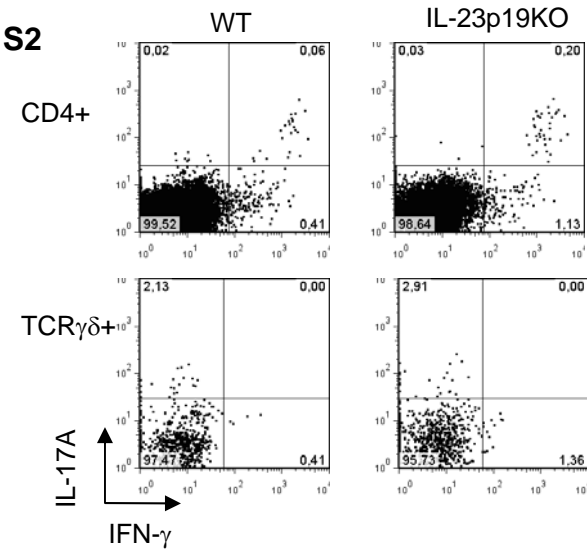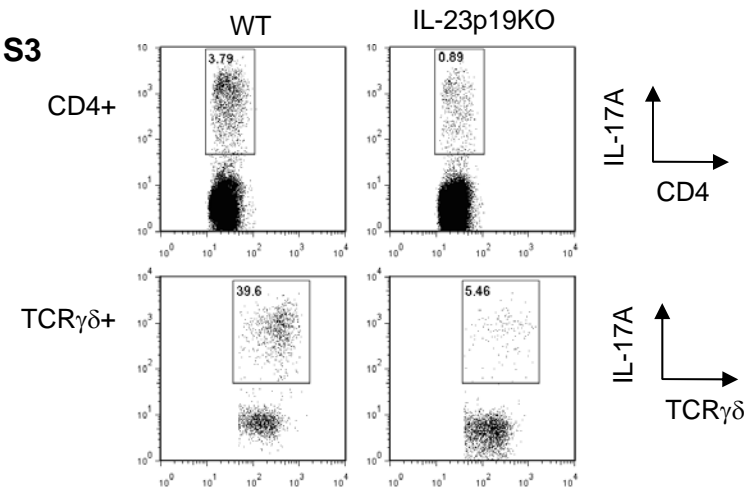

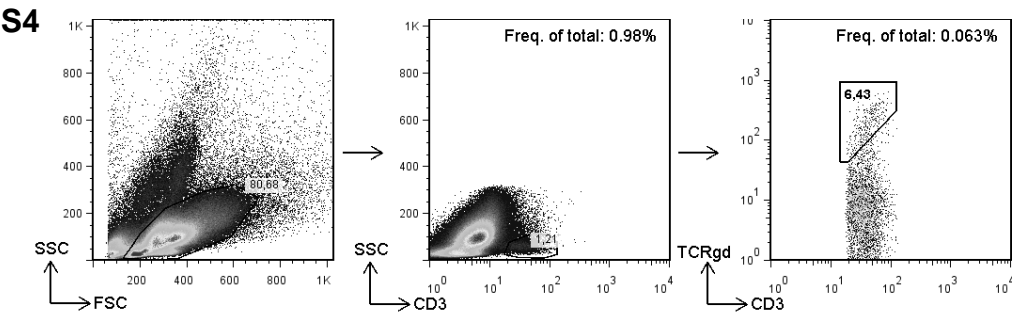

Supplement: Additional file 1 — An Adobe PDF file containing four supplemental figures. Figure S1: Antigen-induced arthritis was induced in WT and IL-23p19KO mice and at day 7 after i.a. mBSA injection the splenocytes were isolated and stimulated for four hours with PMA/Ionomycin and analyzed by flow cytometry. Shown are total ungated cells. Figure S2: Splenocytes were isolated from naïve WT and IL-23p19KO mice and stimulated for four hours with PMA/Ionomycin and analyzed by flow cytometry for intracellular expression of IL-17A and IFN-γ. CD4+ (top panel) and CD3+TCRγδ+ (lower panel) T cells were gated. Numbers indicate percentage of cytokine-positive cells within each quadrant. Figure S3: Antigen-induced arthritis was induced in WT and IL-23p19KO mice and at day 7 after i.a. mBSA injection cells from the draining lymph-nodes were isolated and stimulated for four hours with PMA/Ionomycin and analyzed by flow cytometry. CD4+ (top panel) and CD3+TCRγδ+ (lower panel) T cells were gated. Numbers indicate percentage of positive cells within each gate. Figure S4: Pseudocolor plot of cells isolated from the joint of a representative WT mouse at day 7 of AIA. Shown is the FSC/SSC of all cells (left figure), and subsequent gating-steps for plotting TCRγδ+ T cells. Numbers adjacent to gates indicate the percentage of cells in that specific gate and the numbers in the top-right corner indicate the percentage of cells in the gate relative to all cells. [file ar2893-S1.PDF]
